# TIM-3 blockade reverses oncolytic vaccinia virus-induced DCs inactivation and T cells exhaustion to improve antitumor immunity and therapeutic efficacy

**DOI:** 10.1186/s13046-025-03596-0

**Published:** 2025-11-25

**Authors:** Peipei Ye, Yi Wu, Xue Yang, Hui Wu, Yongming Xia, Gongqiang Wu, Gang Cheng, Weidong Sun, Junyu Zhang, Shibing Wang, Xiangmin Tong

**Affiliations:** 1 Zhejiang Key Laboratory of Zero Magnetic Medicine, Affiliated Hangzhou First People’s Hospital, School of Medicine, Westlake University, Hangzhou, Zhejiang China; 2 Department of Hematology, The Affiliated People’s Hospital of Ningbo University, Ningbo, Zhejiang China; 3 Department of Clinical Research Center, Affiliated Hangzhou First People’s Hospital, School of Medicine, Westlake University, Hangzhou, Zhejiang China; 4 School of Pharmaceutical Sciences, Zhejiang Chinese Medical University, Hangzhou, Zhejiang China; 5 School of Basic Medical Sciences and Forensic Medicine, Hangzhou Medical College, Hangzhou, Zhejiang China; 6 Department of Hematology, Yuyao People’s Hospital, Yuyao, Zhejiang China; 7 Department of Hematology, Affiliated Dongyang Hospital of Wenzhou Medical University, Dongyang, Zhejiang China; 8 Department of Stomatology, Zhejiang Provincial People’s Hospital (Affiliated People’s Hospital, Hangzhou Medical College), Hangzhou, Zhejiang China; 9 Department of Hematology, Shaoxing Central Hospital, Shaoxing, Zhejiang China; 10 Department of Hematology, The Fifth Affiliated Hospital of Wenzhou Medical University, Lishui Central Hospital, Lishui Hospital of Zhejiang University, Lishui, Zhejiang China; 11 Department of Clinical Laboratory, Affiliated Hangzhou First People’s Hospital, School of Medicine, Westlake University, Hangzhou, Zhejiang China

**Keywords:** Oncolytic vaccinia viruses, TIM-3, DCs, CD8^+^ t cells, Immunotherapy

## Abstract

Oncolytic vaccinia viruses (OVV) demonstrate the capacity to inflame the tumor microenvironment (TME) and elicit infiltrating tumor-specific T cells responses. However, OVV treatment paradoxically alters the cancer-immune equilibrium within tumors, attenuating anti-tumor immunity and necessitating a deeper understanding of the viral-induced immune landscape to optimize therapeutic potential. This study investigates the impact of single-domain antibody-armed OVs targeting TIM-3 on TME remodeling, aiming to overcome localized immunosuppression and enhance tumor responsiveness to immunotherapeutic interventions. We engineered a tumor-selective OVV vector encoding single-domain antibodies against murine (mNbTIM3) or human (hNbTIM3) TIM-3. The therapeutic efficacy of OVV-mNbTIM3 was evaluated across multiple murine cancer models, demonstrating that localized V_HH_TIM3 delivery via OVV-mNbTIM3 promotes systemic anti-tumor immunity in established cancers. Mechanistically, OVV-mNbTIM3 treatment enhances DCs maturation and tumor-specific CD8^+^ T cells activation. Notably, intratumoral expression of hNbTIM3 also conferred therapeutic benefit in humanized mice bearing patient-derived cancer xenografts. These findings provide mechanistic insights for enhancing OVV combination therapies and inform the rational design of next-generation oncolytic viruses with engineered immunomodulatory properties.

**Supplementary Information:**

The online version contains supplementary material available at 10.1186/s13046-025-03596-0.

## Introduction

Cancer remains a paramount global health challenge, currently ranking as the second leading cause of mortality worldwide [1]. Over recent decades, cancer immunotherapies, including monoclonal antibodies, adoptive cell therapies, immune checkpoint inhibitors, and oncolytic viruses (OVs), have emerged as transformative therapeutic modalities capable of inducing durable clinical responses across diverse malignancies, thereby reshaping oncological treatment paradigms [2–6].

Among OV platforms, vaccinia virus has emerged as a particularly promising candidate due to its robust replicative capacity, intrinsic immunogenicity, and extensive preclinical validation [7, 8]. However, despite encouraging preclinical efficacy and ongoing clinical investigation, oncolytic vaccinia virus (OVV) therapies frequently demonstrate suboptimal antitumor activity in human trials [9, 10]. This therapeutic limitation stems from multiple biological barriers, including antiviral immune clearance, off-tumor tropism, and the paradoxical induction of immunosuppressive tumor microenvironment (TME) dynamics [8, 11]. While antiviral and pharmacokinetic challenges have been partially addressed through viral engineering strategies [12], the immunomodulatory consequences of OVV administration, particularly the induction of immunosuppressive feedback loops, remain poorly characterized [13]. This knowledge gap hinders the rational development of combination regimens to augment OVV efficacy.

In the present study, we demonstrate that while intratumoral OVV therapy induces initial tumor regression, it concurrently drives functional exhaustion of CD8^+^ T cells within the TME. This dysfunctional phenotype is marked by sustained upregulation of the inhibitory receptor TIM-3 (T cells immunoglobulin and mucin domain-containing protein 3), co-expression of multiple exhaustion-associated markers, and impaired cytotoxic effector function. Critically, TIM-3^+^CD8^+^ T cells exhibit reduced proliferative capacity and diminished production of antitumor cytokines (IFN-γ, TNF-α), correlating with incomplete therapeutic responses and disease relapse [14]. These findings elucidate a previously unrecognized mechanism of OVV resistance and provide a rationale for targeting TIM-3 signaling pathways to restore T cells functionality in combination immunotherapy regimens.

To enhance the therapeutic efficacy of OVV, current strategies predominantly utilize combinatorial approaches with immune checkpoint inhibitors [15, 16], cytokines [17, 18], adoptive cell therapies [19, 20], or chemotherapeutic agents such as cyclophosphamide [9, 21]. Alternatively, genetic engineering of the viral backbone aims to mitigate intrinsic limitations [12]. While these “dual-modality” strategies demonstrate promise, a more rational paradigm involves first delineating the mechanistic pathways of immunosuppression induced by OVV monotherapy, then strategically counteracting these barriers through targeted interventions or viral redesign [22–24].

TIM-3, initially characterized as a marker of IFN-γ-producing effector T cells, has emerged as a critical immune checkpoint regulating adaptive immune exhaustion [25]. Clinical investigations of anti-TIM-3 monoclonal antibodies are underway across multiple malignancies, given its correlation with T cells dysfunction in chronic antigen exposure settings [26, 27]. Within the TME, sustained TIM-3 expression on CD8^+^ T cells serves as a hallmark of dysfunctional differentiation, characterized by impaired cytokine production and cytotoxic activity [28]. Notably, TIM-3 signaling extends beyond T cells regulation to modulate DCs function, where its blockade inhibits DCs tolerance programs and preserves CD8^+^ T cells effector functionality [29].

Building on our observation of OVV-driven expansion of exhausted TIM-3^+^CD8^+^ T cells in the TME, we hypothesized that TIM-3 neutralization could synergize with OVV therapy by reprogramming immunosuppressive networks. Using preclinical solid tumor models and in vitro co-culture systems, we demonstrate that systemic TIM-3 blockade reverses T cells exhaustion induced by OVV, restores antigen-specific cytotoxicity, and enhances intratumoral immune infiltration. To translate this mechanistic insight, we engineered a recombinant OVV variant (OVV-NbTIM3) encoding a single-domain nanobody targeting murine/human TIM-3. This genetically modified virus demonstrated equivalent therapeutic efficacy to combination regimens in immunocompetent murine models, with superior safety profiles compared to systemic antibody administration. Notably, OVV-NbTIM3 retained oncolytic potency while inducing sustained reprogramming of regulatory T cells (Tregs) and tumor-associated macrophages (TAMs), thereby creating a permissive TME for adaptive immune activation.

Collectively, these findings establish a mechanistic paradigm for OVV optimization through targeted viral engineering to neutralize adaptive resistance mechanisms. The dual-pronged strategy of OVV-NbTIM3, combining direct tumor lysis with checkpoint neutralization, offers a clinically translatable platform to address immune evasion challenges in viro-immunotherapy.

## Materials and methods

### Cell lines

The cell lines employed in this study were obtained from the American Type Culture Collection (ATCC, USA), including HEK293 cells (human embryonic kidney cells), Hela-S3 (Human cervical cancer cells), Raji cells (human lymphoma cells), MDA-MB-231 cells (human triple negative-breast carcinoma), and 4T1 cells (mouse mammary carcinoma cells), A20 (mouse lymphoma cells), Panc02 (mouse pancreatic carcinoma cells), CT26 (mouse colon carcinoma cells). These cell lines were cultured in Dulbecco’s modified Eagle’s medium (DMEM, Gibco-Thermo Fisher Scientific, USA) supplemented with 10% fetal bovine serum (FBS, Gibco). Specifically, Hela-S3 cells were kept in suspension in a spinner flask in serum-free medium (H740KJ, Basalmedia, Shanghai, China). All cells were grown in an incubator set at 37 °C and 5% CO_2_.

### Oncolytic vaccinia viruses

GenScript (Nanjing, China) produced the mV_HH_TIM-3 and hV_HH_TIM-3 gene fragment, which was then subcloned into the shuttle plasmid pVV-Control to create the recombinant plasmid pVV-mV_HH_TIM-3 or pVV-hV_HH_TIM-3. The following experimental steps of packaging, amplification, purification, and titer determination of oncolytic vaccinia virus were performed according to previously described procedures [8].

### Western blot analysis

A20 or Raji cells were seeded at a density of 5 × 10^5^ cells per well in 6-well plates and infected with OVV-mNbTIM3, OVV-hNbTIM3 or OVV control at a MOI of 1. After 48 h, the cell lysates and supernatant were collected, and 10 µL of them were mixed in equal amounts with a 2×loading buffer (P0015, Beyotime, Shanghai, China). The following experimental steps of Western blotting were performed according to previously described procedures [30]. The antibodies used were anti-Flag antibody (Abcam, ab205606) and goat anti-mouse IgG (H + L) (Invitrogen, 31430).

### Crystal violet staining

4T1 and MDA-MB-231 cells were seeded in 6-well plates at a density of 5 × 10^4^ cells per well, respectively, and cultured in an incubator at 37 °C with 5% CO_2_ atmosphere. When the tumor cells reached 70% confluence, OVV-mNbTIM3 or OVV-hNbTIM3 and OVV control at MOIs of 0, 0.001, 0.01, 0.1, 1, and 10 were introduced to the wells. The supernatants were removed after 72 h of incubation, and the 0.2% (w/v) crystal violet solution (C0121, Beyotime) was added to the wells for staining. After 5 min, the staining solution was taken from the wells and washed five times with ddH_2_O. A scanner was used to capture the image.

### Viral oncolysis and replication

A total of 5 × 10^3^ 4T1, A20, Panc02, CT26, Raji and MDA-MB-231 cells were seeded into 96-well plates and cultured overnight prior to treatment with different oncolytic vaccinia virus for 48 h. Cell viability was evaluated by CCK-8 assays. The TCID50 assay was used to calculate the replication of the oncolytic vaccinia virus, Briefly, cells were seeded into 24-well plates and infected with different oncolytic vaccinia virus. The medium containing viruses was removed, and fresh medium was added 2 h after infection. Cells were harvested at serial time points as indicated (48, 72, and 96 h after infection). Then, the vaccinia virus titers were measured by TCID50 assay.

### Single-cell RNA sequencing

The data from single-cell RNA sequencing comes from our previous study [8]. In brief, 4T1 cells were injected into the right flank of BALB/c mice, when the tumor reached 50 mm3, 3 doses of 5 × 10^7^ pfu of OVV were injected i.t. PBS injected mice served as controls. The tumors were then dissociated into single cells and the following experimental steps of scRNA-seq were performed according to previously described procedures.

### BMDC and T-cell isolation

Bone marrow cells were collected from the tibias and femurs of C57BL/6 mice and cultured with complete RPMI-1640 medium containing 20 ng/ml mGM-CSF (PeproTech). Fresh medium supplemented with mGMCSF was added to the culture 3 days later. Immature BMDCs were collected and ready to use on day 7. Naïve T cells were isolated from the spleen with a negative CD3^+^ T-cell isolation kit (STEMCELL Technologies).

### Measurement of cytokines

In the experiment of studying the activation of DCs function by anti-TIM-3, after 24 h of infection with OVV-mNbTIM3 or OVV (1MOI) in A20 cells, the supernatant was collected and added to mouse DCs. After co incubation for 48 h, ELISA MAX Standard Sets of IL-12 (433604, Biolegend), TNF-α (430901, Biolegend) and IL-1β(432616, Biolegend) were used to determine the concentration of the corresponding cytokines. The measurement method was based on the manufacturing protocol. In the experiment to verify that OVV-mNbTIM3 can induce tumor specific immune memory, after previously cured mice rechallenge experiment, the mice were sacrificed after anesthesia to obtain spleens and prepare splenocytes. The cultured A20, 4T1, Panc02 and CT26 cells were seeded into a 6-well plate at the density of 2 × 10^5^ cells per well, and then 1 × 10^6^ splenocytes were added to make the ratio of splenocytes to tumor cells 5:1. Samples were centrifuged at 600 g for 5 min at RT and the supernatants were collected after 48 h co-culture. ELISA MAX Standard Sets of IFN-γ (430801, Biolegend), TNF-α (430901, Biolegend) and IL-2(431001, Biolegend) were used to determine the concentration of the corresponding cytokines. The measurement method was based on the manufacturing protocol.

### Binding assays

To detect whether the secreted mNbTIM3 or hNbTIM3 could bind to the recombinant mouse TIM3 protein (Cat# TI3-M5252, Acro) or human TIM3 protein (Cat# TM3-H5258, Acro), a FLAG-linked Elisa assay was performed. Briefly, 96-well plates were coated with mouse or human TIM3 protein at a concentration of 10 mg/ml. Then, supernatants of OVVs-infected cells were collected and added to the coated wells and incubated at 4 °C for 12 h. After that, the cell culture supernatant was removed, washed 3 times with 1 × TBS buffer, NbTIM3 binding TIM3 protein was determined by ELISA assays using anti-FLAG M2-HRP antibody (Cat#A8592, Sigma Aldrich).

Binding assays were also performed with TIM3^+^ cells. Mouse CD8^+^ T cells and DCs were sorted out from A20 tumors using CD8 or CD11c antibodies. Human T cells or DCs were sorted out from surgical resection of tissue in tumor patients using flow cytometry. The OVV-mNbTIM3 or OVV-hNbTIM3-infected supernatants were incubated on ice for one hour with target cells (1 × 10^5^). Using flow cytometry and the APC anti-DYKDDDDK Tag antibody (Cat# 637308, Biolegend), mNbTIM and hNbTIM3 binding was assessed.

### Animal experiments

BALB/c mice and C57BL/6 mice were purchased from the Model Animal Research Center of Nanjing University (Nanjing, China). For the establishment of subcutaneous tumor models, the A20, Panc02, 4T1 and CT26 tumor cells were implanted subcutaneously into the right flank of the mice. When the tumor reached approximately 50 to 100 mm^3^, the mice were randomly grouped and intratumorally treated with OVV-mNbTIM3, OVV or PBS. Tumor diameters were measured every two or three days and the tumor volume was calculated by the formula 0.5×length×width^2^. When the tumor volume reached 2000 mm^3^, mice were euthanized. For the combination therapy with OVV and mNbTIM3, bilateral 4T1 and A20 models were used. 4T1 or A20 cells were subcutaneously inoculated into the right and left flanks of mice at the same time. When tumors reached about 50 mm^3^, unilateral intratumoral treatment commenced as above. For the combination therapy with OVV-mNbTIM3 and anti-mouse PD-1 antibody (Clone RMP1-14, BioXCell) or anti-mouse CTLA-4 antibody (Clone 9D9, BioXCell), a subcutaneous 4T1 model was established as previously described. Each mouse was injected intraperitoneally with 200 µg of PD-1 or CTLA-4 antibody, which was initiated from the next day of last viral treatment. For CD8^+^ T cells, CD4^+^ T cells, macrophage or DCs depletion, BALB/c mice were intraperitoneally injected with 500 mg of anti-CD8α antibody (clone 53 − 6.7, Bioxcell), anti-CD4 antibody (clone GK1.5, Bioxcell), anti-CSF1R antibody (clone AFS98, Bioxcell) or anti-CD317 antibody(clone 927, Bioxcell) every other day for a total of three times at related time points (*n* = 10 mice/group).

For PBMC-humanized CDX model, exponentially growing MDA-MB-231 or Raji cells were harvested and then injected subcutaneously into the flanks of NCG mice. PBMCs were inoculated into the mice next day. When tumors reached about 50 mm^3^, unilateral intratumoral treatment commenced as above.

For PBMC-humanized PDX model, the breast tumor of patients diagnosed with TNBC was maintained on ice and brought to the laboratory within 1 h, after which they were harvested and dissociated into single cells and/or organoids by mechanical mincing and digestion. Cells were filtered through a 70 mm sterile filter. All the viable tumor cells were resuspended in 50% volume Matrigel and injected into the fourth mammary fat pads of NCG mice. When tumors reached about 50 mm^3^, unilateral intratumoral treatment commenced as above.

### Flow cytometry

Biolegend provided the following antibodies: PE anti-mouse CD45 (Clone 30-F11), APC anti-mouse CD3 (Clone 17A2), FITC anti-mouse CD4 (Clone GK1.5,), PE anti-mouse CD4 (Clone GK1.5), FITC anti-mouse CD8α (Clone 53 − 6.7), APC/Fire 750 anti-mouse IFN-γ (Clone XMG1.2), Biotin anti-mouse CD107a (Clone 1D4B), APC anti-mouse/human CD11b (Clone M1/70), Percp anti-mouse F4/80 (Clone BM8), Percp anti-mouse CD86 (Clone GL-1), APC anti-mouse CD80 (Clone 16-10A1), FITC anti-mouse CD206 (Clone C068C2), PE/Fire 700 anti-mouse CD206 (Clone C068C2), PE/Cyanine7 anti-mouse Tim-3 (Clone RMT3-23), PE/Cyanine7 Mouse IgG1 (Clone MOPC-21). For the preparation of single-cell suspensions of tumor tissues, mice were anesthetized and sacrificed, and the tumor tissues were harvested and placed in serum-free medium (Basalmedia) with 0.2% collagenase IV (Sigma-Aldrich, Germany). The tumor tissues were then cut into 1–2 mm pieces, digested for 2 h, and passed through 70-µm nylon filters (Jetbiofil, Guangzhou, China) to obtain single-cell suspensions. Then, collagenase was removed by centrifugation and the cell pellets were suspended in serum-free medium and adjusted to 2 × 10^7^ cells/mL. Data analysis was performed using FlowJo software (TreeStar; OR, USA).

### Statistical analyses

For all statistical studies, Prism 8.2.1 (GraphPad Software, California, USA) was utilized. The statistical differences between the groups were investigated using the analysis of variance. The Kaplan-Meier method was used to generate the survival curve, and the Log-Rank test was used to assess the statistical significance of the differences between the groups. In every statistical analysis, a value of *P* < 0.05 was considered statistically significant.

## Results

### Oncolytic efficacy is restricted by upregulation of TIM3 on tumor-infiltrating T cells

**Fig. 1:**
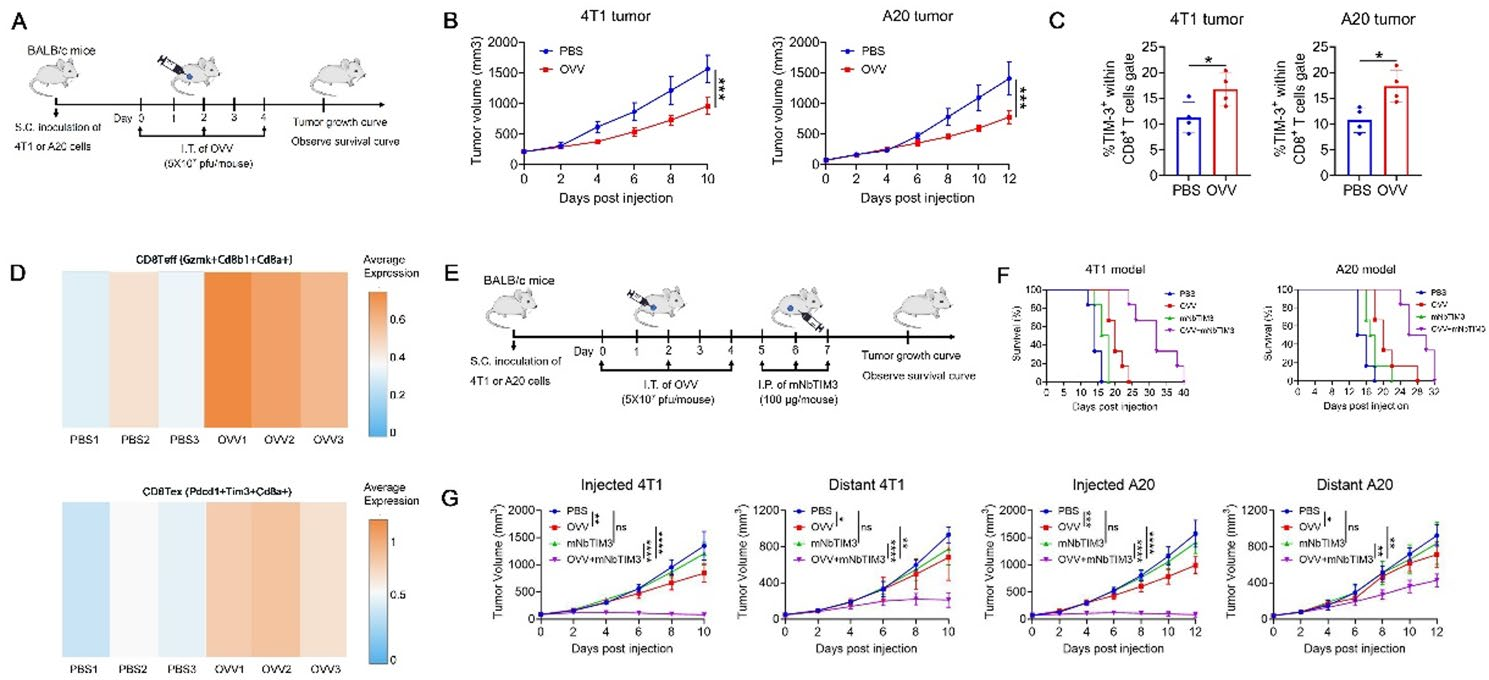
TIM-3 upregulation in the tumor microenvironment restricts the antitumor responses of OVV. **A** Schematic representation of experimental design and treatment timeline. **B** 4T1 or A20-tumor-bearing WT mice were administered OVV or PBS. *n* = 6 for 4T1 model and *n* = 7 for A20 model. **C** Tumor-infiltrating TIM-3^+^CD8^+^ T cells isolated from PBS-treated and OVV-treated tumors one day after the last virus injection were detected by flow cytometry. **D** Tumor-infiltrating effective CD8^+^ T cells (CD8Teff) and exhaustive CD8^+^ T cells (CD8Tex) from our previous scRNA sequencing data. **E** Treatment scheme for combination therapy of OVV and mNbTIM3 in bilateral 4T1 and A20 models. **F** Survival of mice cured by combination therapy (*n* = 6). **G** Growth of injected tumors and distant tumors in bilateral 4T1 and A20 model (*n* = 6). The data are shown as the means ± SD. ns, no significant difference; *p < 0.05, **p < 0.01, ***p < 0.001, ****p < 0.0001

In our previous study, we developed an immunotherapeutic OVV that showed potent and durable antitumor effects [8, 30]. To further enhance the anticancer effect of OVV, we evaluated the antitumor efficacy of OVV in a syngeneic 4T1 tumor and A20 model (Fig. 1A). OVV therapy significantly inhibited tumor but did not completely eradicate the tumors (Fig. 1B). While OVV remodeled the TME by enhancing the recruitment and activation of CD8^+^ T cells, it concomitantly increased the expression of TIM3 (Figs. 1C, D), which hindered the anticancer effects of cytotoxic T cells. Therefore, we sought to combine OVV virotherapy and anti-TIM-3 immunotherapy to compensate for their respective weaknesses. The combination of an anti-mouse TIM3 single domain antibody (mNbTIM3) and OVV restrained not only virus-injected tumors but also distant tumors in the 4T1 and A20 bilateral tumor model (Fig. 1E). Mice treated with combination immunotherapy showed remarkably better overall survival than those treated with monotherapy (Fig. 1F). The combination therapy more potently suppressed the growth of both treated tumors and distant untreated tumors than OVV or anti-TIM3 monotherapy (Fig. 1G).

### mNbTIM3 induces DCs maturation and T cells activation during OVV treatment

**Fig. 2:**
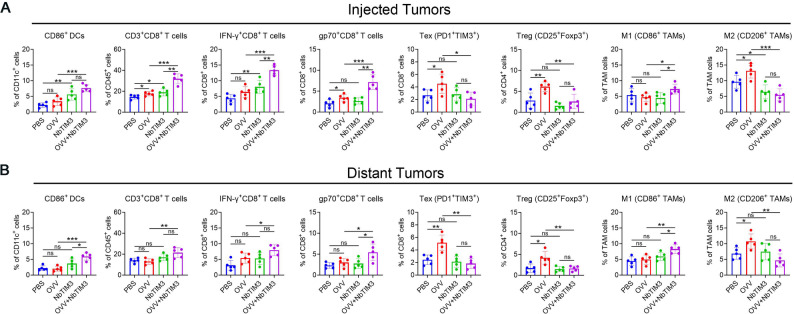
TIM-3 blockade significantly enhances the OVV-induced antitumor immunity in A20 tumor models. Two days after last injection of OVV and mNbTIM3 in combination into the tumors in the right flank (injected tumors), TILs in injected tumors (**A**) and distant tumors (**B**) were analyzed by flow cytometry (*n* = 5). The data are shown as the means ± SD. ns, no significant difference; *p < 0.05, **p < 0.01, ***p < 0.001

We hypothesized that mNbTIM3 orchestrates immune infiltration in the TME to improve the antitumor effect of OVV. Consistent with this hypothesis, we evaluated the infiltration of immune cells after combination therapy of OVV and mNbTIM3 in the A20 bilateral tumor model. The results show that combination therapy increase in local tumor infiltration with more mature CD86^+^ DCs, activated IFN-γ^+^CD8^+^ T cells and tumor antigen-specific T cells (gp70-tetramer^+^ CD8^+^ T cells) compared to the OVV or mNbTIM3 treated mice (Fig. 2A). Further, we found that OVV treatment led to an expansion of PD-1^+^TIM3^+^CD8^+^ T cells, CD25^+^Foxp3^+^ Tregs and downregulation of CD86 and upregulation of CD206 in TAMs from the OVV-treated mice compared with the PBS and mNbTIM3-treated mice. Surprisingly, the OVV-induced immunosuppressive TME could be reprogrammed by intraperitoneal injection of mNbTIM3. OVV combined with mNbTIM3 caused a notable loss of Texs and Tregs compared with the OVV treatment (Fig. 2A). Identical changes in immune status were observed in the noninjected contralateral tumors, characterized by infiltration of the same immune cells as those seen in the injected right-sided tumors (Fig. 2B).

We also carried out depletion experiments to analyze the key immune cells involved in the combination therapy and discovered that DCs and CD8^+^ T cells played a predominant role in the response to the combination therapy (Figure S1). Taken together, these results indicate that combination therapy incorporating OVV and anti-TIM3 therapy can overcome resistance to immunotherapy in immunosuppressive TME, resulting in enhanced anticancer effects.

### mNbTIM3-armed oncolytic vaccinia virus improves antitumor activity

**Fig. 3:**
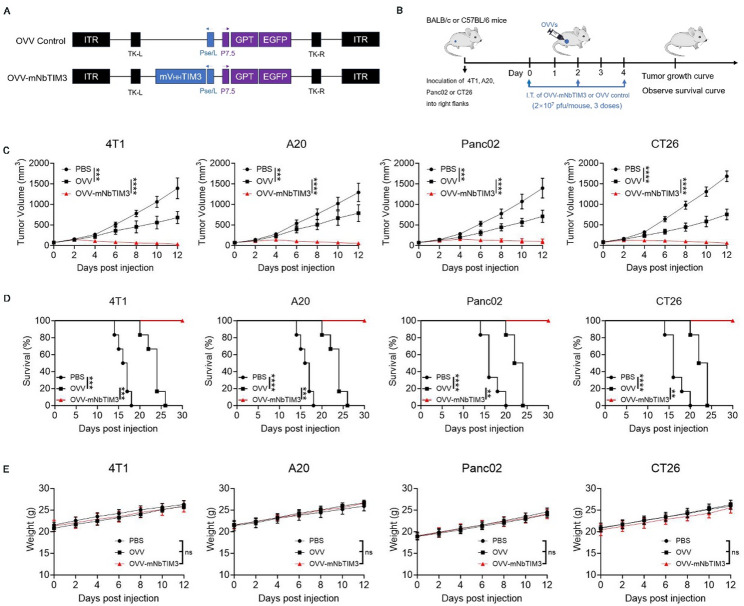
Intratumoral injections of OVV-mNbTIM3 enhances antitumor efficacy in subcutaneous tumor models. **A** Schematic diagram of a recombinant oncolytic vaccinia virus expressing mNbTIM3. **B** Schematic representation of experimental design and treatment timeline. 4T1, A20, Panc02 or CT26 tumor-bearing mice were administered different immunotherapies or PBS (*n* = 6 biological replicates). **C** Tumor growth. **D** Survival. **E** Body weight. These data are shown as the means ± SD. ns, no significant difference; *p < 0.05, **p < 0.01, ***p < 0.001, ****p < 0.0001

Through homologous recombination, we generated a recombinant oncolytic vaccinia virus, OVV-mNbTIM3, with a gene fragment of anti-mouse TIM3 nanobody in the backbone of a thymidine kinase (TK)-destroyed WR-VV (Fig. 3A). We evaluated the antitumor activity of the OVV-mNbTIM3 in subcutaneous tumor models (mouse breast cancer, 4T1; mouse lymphoma A20; mouse pancreatic cancer, Panc02; mouse colorectal cancer, CT26) in immunocompetent BALB/c or C57BL/6 mice (Fig. 3B). In all four models, mice treated with OVV-mNbTIM3 had significantly lower tumor volume (Fig. 3C) and prolonged survival compared with mice treated with OVV or PBS (Fig. 3D). Five-sixth of OVV-mNbTIM3-treated mice achieved tumor complete remission (CR) in 4T1, A20 and Panc02 model, and Six-sixth of the OVV-mNbTIM3-treated mice achieved CR in CT26 model, while none of the six mice treated with PBS or OVV control achieved CR (Figure S2). There was no significant difference in body weight among the three groups of mice (Fig. 3E). The results showed that the recombinant oncolytic vaccinia virus OVV-mNbTIM3 can effectively exert superior antitumor activity in vivo.

In addition, we analyzed the ability and biosafety of OVV combined with NbTIM3 nanobodies and recombinant oncolytic acne virus OVV-mNbTIM3 to express NbTIM3 nanobodies. The safety of the two treatment methods was evaluated by HE staining and blood routine, and the results are shown in Figure S3 A and B, both treatment methods did not cause significant damage to the liver and kidney tissues of mice. Next, we analyzed the ability of two treatment methods to express NbTIM3 in mice using western blot and IHC. The results are shown in Figures S3 C and D, the nanoparticles produced by OVV-mNbTIM3 are mainly expressed at the tumor site and do not decay rapidly. On the contrary, they first exhibit logarithmic growth and then reach higher levels. However, over time, some nanoparticles that may diffuse into the bloodstream may be captured by the liver, leading to lower levels of reduction. This has a more lasting effect than intraperitoneal injection of NbTIIM3 antibody.

Meanwhile, the mNbTIM3 was efficiently expressed from OVV-mNbTIM3-infected A20 cells as detected by western blot assay (Figure S4A). To confirm the binding ability of secreted mNbTIM3 to TIM-3, we incubated mouse T cells with the supernatant of OVV-mNbTIM3-infected cells, followed by the addition of anti-FLAG antibody. Flow cytometric analyses showed that mNbTIM3 was able to bind to surface TIM3 on mouse DCs and CD8^+^ T cells (Figure S4B). Furthermore, the secretion of the CD19BiTE was quantified from the supernatants of OVV-CD19BiTE infected cells by ELISA assay. The crystal violet staining and MTT assay showed that both OVV-mNbTIM3 and OVV showed dose (MOI)-dependent oncolytic activity against murine tumor cells. Both crystal violet staining and MTT assay confirmed that there was no statistical difference in the oncolytic activity between OVV-mNbTIM3 and OVV (Figure S4C, D). Moreover, OVV-mNbTIM3 and OVV showed similar replication characteristics in murine tumor cell lines (Figure S4E). These data indicated that the replication and oncolytic ability of the recombinant OVV was not affected by the mNbTIM3 transgene.

### Enhanced antitumor immunity by OVV-NbTIM3 in subcutaneous tumor models

**Fig. 4:**
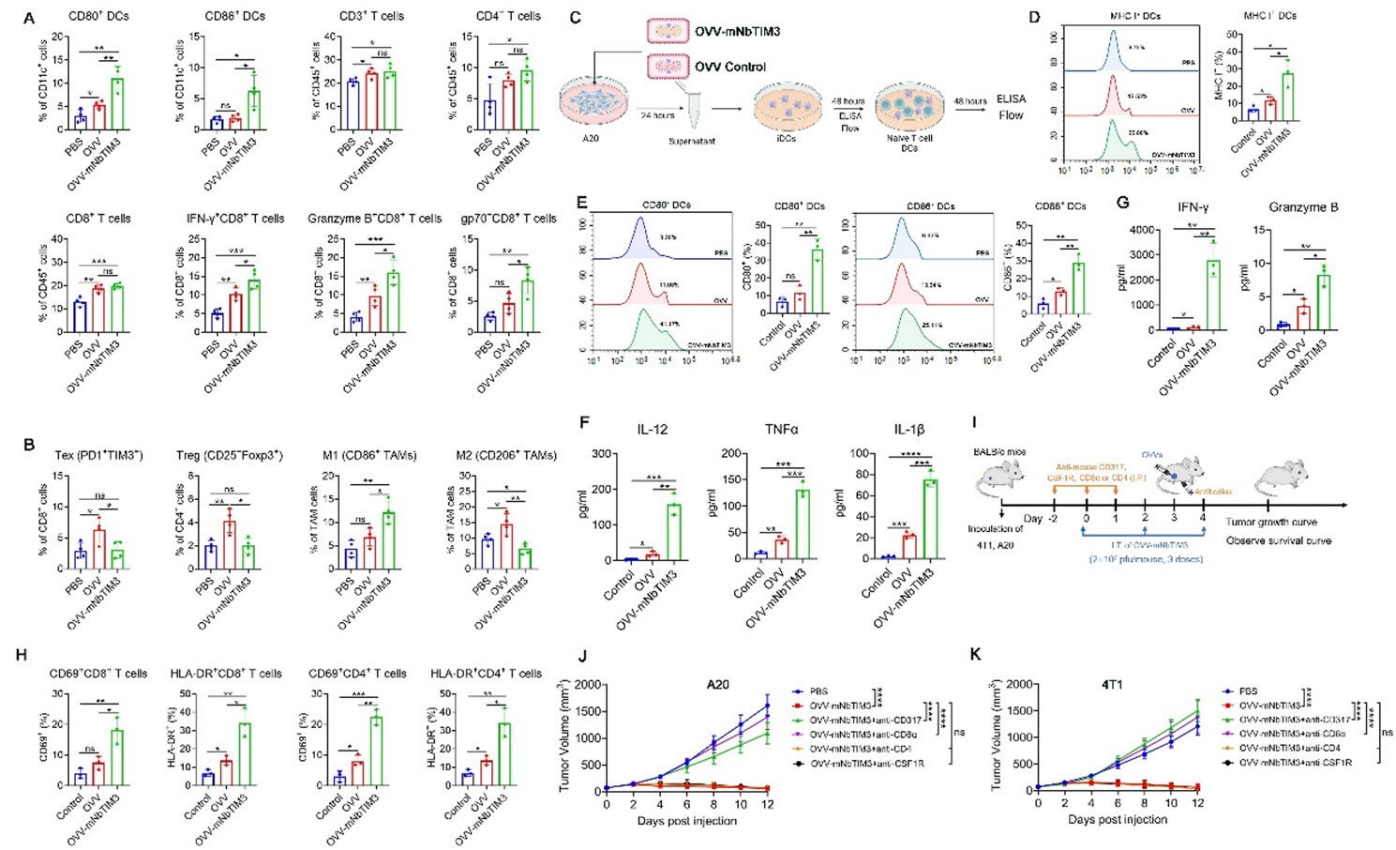
OVV-mNbTIM3 reshapes the tumor immune microenvironment through DCs and CD8^+^ T cells. (A-B) A20 models were established as previously described and the single-cell suspensions were preprepared two days post the last viral injection. **A** Flow cytometric analysis of the proportions of CD80^+^ DCs, CD86^+^ DCs, CD3^+^ T cells, CD8^+^ T cells, CD4^+^ T cells, IFN-γ^+^CD8^+^ T cells, Granzyme B^+^CD8^+^ T cells and gp70^+^CD8^+^ T cells in A20 tumors. **B** Flow cytometric analysis of the proportions of Tex (PD-1^+^ TIM-3^+^CD8^+^ T cells), Treg (CD25^+^ FoxP3^+^CD4^+^ T cells), M1 (CD80^+^F4/80^+^CD11b^+^ Macrophages) and M2 (CD206^+^F4/80^+^CD11b^+^ Macrophages) in A20 tumors. **C** Diagrammatic sketch of the coculture experiment performed ex vivo. A20 cells were treated with OVV-mNbTIM3 or OVV control at MOI of 1 on day 0, and then the supernatant of each group was collected and fed to immature DCs (iDCs) on day 2. One batch of DCs was used to detect maturation markers by flow cytometry and secreted cytokines by ELISA on day 4. The other batch of DCs was cocultured with naive T cells on day 4, and T-cell activation was evaluated on day 8. MHC I (**D**) and CD80, CD86 (**E**) expression on DCs fed the indicated supernatants was detected by flow cytometry on day 4. *n* = 3. **F** IL-12, TNF-α, and IL-1β levels in the culture medium of DCs fed with the indicated supernatants were determined by ELISA on day 4. *n* = 3. **G** IFN-γ and Granzyme B concentrations were determined by ELISA in the DCs plus T-cell coculture system on day 8. *n* = 3. **H** CD69, and CD44 expression on CD4^+^ T cells and CD8^+^ T cells was evaluated on T cells in the DCs plus T-cell coculture system on day 8. *n* = 3. **I** Experimental schematic of mice: tumor-bearing mice were administered OVV-mNbTIM3 and treated with intraperitoneal injections of anti-CD317, anti-CD8α, anti-CD4 or anti-CSF1R. Tumor growth of A20 (**J**) and 4T1 (**K**) tumor-bearing mice (*n* = 8 for A20 models, (*n* = 6 for 4T1 models). Data are shown as the mean ± SD. ns, no significant difference; *p < 0.05, **p < 0.01, ***p < 0.001, ****p < 0.0001

Further, we addressed whether OVV-mNbTIM3 produced immune responses similarly to those produced by OVV combined with mNbTIM3. We observed that intratumoral injection of the OVV-mNbTIM3 significantly increased the composition of CD86^+^ and CD80^+^ in the DCs, gp70^+^, Granzyme B^+^ and IFN-γ^+^ in the CD8^+^ T cells indicating that OVV-mNbTIM3 treatment could effectively promote the maturation of DCs and the activation of tumor specific CD8^+^ T cells in the A20 model (Fig. 4A). Meanwhile, OVV-mNbTIM3 had comparable effects on reprogramming PD-1^+^TIM3^+^CD8^+^ T cells, TAMs and CD25^+^Foxp3^+^ Tregs (Fig. 4B).

To verify whether OVV-mNbTIM3 can promote DCs cell maturation, A20 cells were treated with OVV-mNbTIM3, OVV control, or PBS. Then, we fed immature DCs (iDCs) separately with the supernatant collected from each processing group and detected maturation markers on the DCs surface (Fig. 4C). Compared with the control, the A20 supernatant generated with OVV-mNbTIM3 significantly upregulated the expression of MHC I on DCs, unlike the supernatants obtained from OVV-infected or PBS-treated A20 cells (Fig. 4D). DCs exhibited modestly increased expression of the costimulatory molecules CD80 and CD86 after feeding with the supernatants from OVV-infected, but these levels were far lower than those DCs fed the OVV-mNbTIM3-infected cell supernatant (Fig. 4E). In addition, the DCs supplied with the supernatant from OVV-mNbTIM3-infected tumor cells produced much higher concentrations of IL-12, TNF-α, and IL-1β (Fig. 4F). To determine whether these activated DCs can prime de novo T-cell responses effectively ex vivo, we stimulated iDCs with supernatants from OVV-mNbTIM3-infected A20 cells and then cocultured these DCs with naive T cells. Higher levels of IFN-γ and Granzyme B were detected in the culture medium from wells where T cells were cocultured with DCs stimulated with supernatants from OVV-mNbTIM3-infected A20 cells (Fig. 4G), indicating the activation and increased cytotoxic potential of the T cells. Furthermore, both CD4^+^ T cells and CD8^+^ T cells were primed by DCs stimulated with supernatants from OVV-mNbTIM3-infected A20 cells, as demonstrated by the elevated expression of the activation markers CD69 and HLA-DR (Fig. 4H). All the evidence above illustrated that secretory mNbTIM3 produced by OVV-mNbTIM3 infection effectively restored the ability of DCs to drive effector T cells generation ex vivo.

Given the changes in DCs, macrophage phenotype and the recruitment of CD8^+^ T cells and CD4^+^ T cells induced by OVV-mNbTIM3, to confirm their critical role during treatment, we depleted DCs, macrophages, CD8^+^ T cells or CD4^+^ T cells in A20 or 4T1-bearing mice by anti-CD317, anti-CSF1R, anti-NK1.1, anti-CD8α or CD4 antibodies, respectively (Fig. 4I). By observing tumor growth and survival in mice, we found that depletion of DCs and CD8^+^ T cells impaired the efficacy of OVV-mNbTIM3 treatment (Figs. 4J-K, Figure S5). Altogether, these results indicated that the OVV-mNbTIM3 could reshape the TME by recruiting immune cells and maturing DCs and activating the tumor-infiltrating CD8^+^ T cells.

### Establishment of long-term antitumor memory and increased sensitivity to immune checkpoint inhibitors treatment

**Fig. 5:**
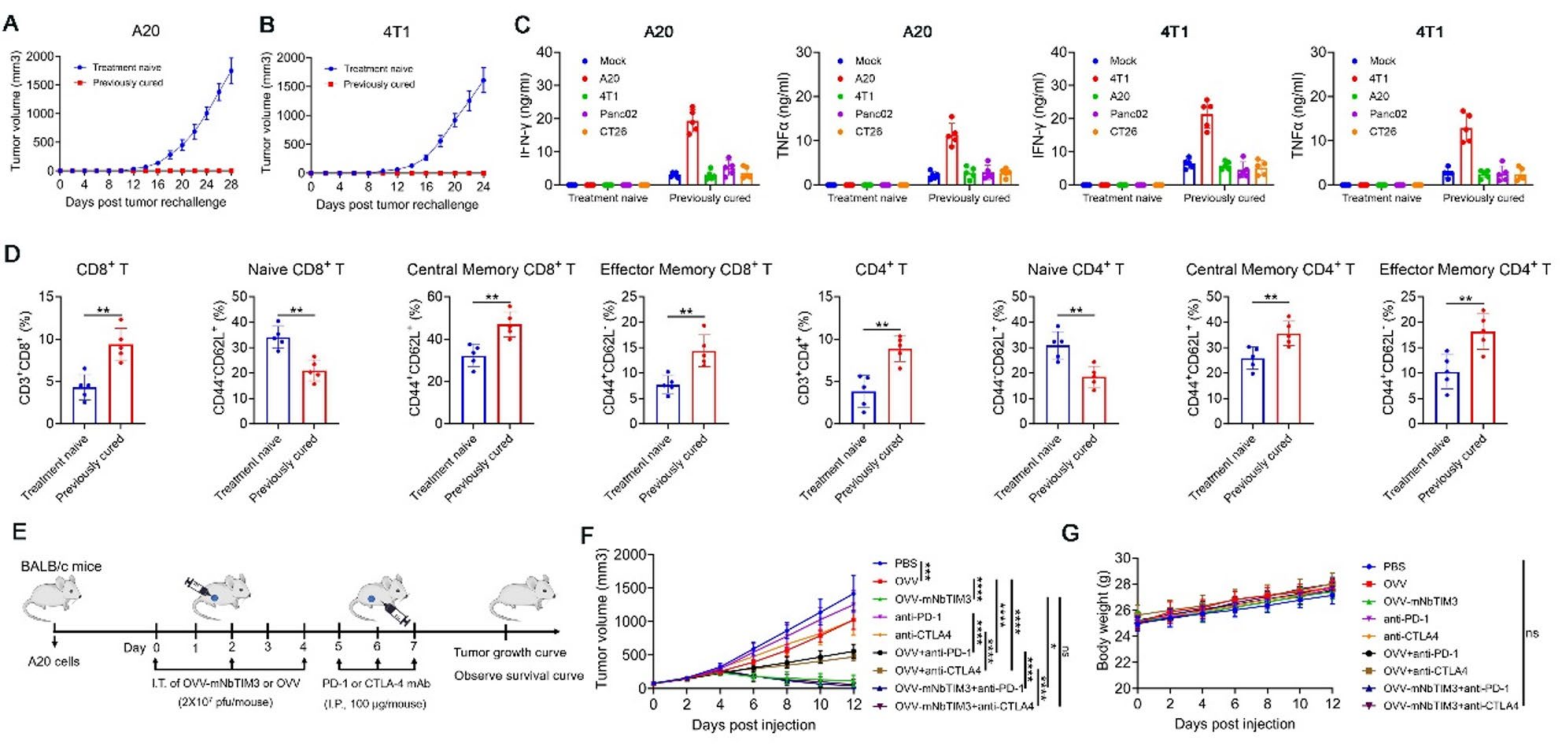
OVV-mNbTIM3 establishes long-term tumor-specific immunological memory and sensitizes lymphoma to Immune checkpoint inhibitors. **A**-**B** Tumor volumes of previously cured mice rechallenged with A20 or 4T1 cells subcutaneously. **C** Co-culture of A20 or 4T1 cells or other mouse cancer cells with splenocytes from the A20 or 4T1 rechallenged mice. The levels of cytokines in the co-culture system were detected by ELISA. **D** At the end of rechallenge, Flow cytometric analysis of the proportion of CD8^+^ T cells and CD4^+^ T cells, naïve (CD62L^+^CD44^−^), central memory (CD62L^+^CD44^+^) and effector memory (CD62L^−^CD44^+^) CD8^+^ T cells and CD4^+^ T cells in splenocytes. **E** Treatment scheme of combination OVV-mNbTIM3 and anti-PD-1 antibody or anti-CTLA-4 antibody. **F** Mean tumor volume of combination therapy of OVV-mNbTIM3 and anti-PD-1 antibody. **G** Mean tumor volume of combination therapy of OVV-mNbTIM3 and anti-CTLA-4 antibody. Data are presented as means ± SD. Data are presented as means ± SD. ns, ns, not significant; **p < 0.01; ****p < 0.0001

We evaluated whether the intratumoral administration of OVV-mNbTIM3 contributes to the establishment of antitumor memory. To examine the long-term persistence of antitumor memory, mice cured of A20 tumors by OVV-mNbTIM3 were rechallenged with the same tumors 45 days after the last treatment. Tumors in the cured mice were completely rejected within 4 weeks, whereas all age-matched tumor-naive control mice developed tumors (Fig. 5A). This protective effect against tumor rechallenge was also observed in the 4T1 model (Fig. 5B). Furthermore, splenocytes from mice in which OVV-mNbTIM3 had caused CR of A20 tumors or 4T1 tumors were collected, and the immune response against different cancer cells was analyzed. These splenocytes of the cured A20 model stimulated with A20 cells secreted higher concentration of IFN-γ and TNFα than did those stimulated with 4T1, Panc02 and CT26 cells, whereas splenocytes of treatment-naive mice showed no detectable response to any stimulation (Fig. 5C), indicating that the intratumoral treatment with OVV-mNbTIM3 generated tumor-specific memory in the splenocytes. Consistently, the proportion of spleen CD8^+^ and CD4^+^ T cells, as well as the respective central memory (CD62L^+^ CD44^+^) and effector memory (CD62L^−^ CD44^+^) subset T cells were increased in cured A20 mice, whereas naïve CD8^+^ and CD4^+^ T cells (CD62L^+^CD44^−^) were significantly decreased compared to those treatment-naïve mice (Fig. 5D).

We hypothesized that altered immune status in the OVV-mNbTIM3-injected tumors, characterized by increases of TILs, would sensitize tumors to immune checkpoint blockade. In the A20 model, anti-PD-1 or anti-CTLA4 antibody was administered with or without previous OVV control or OVV-mNbTIM3 treatment into tumors (Fig. 5E). Monotherapies with anti-PD-1 antibody or anti-CTLA4 antibody showed no or weak efficacy and induced no CR in tumors not pretreated with OVV-mNbTIM3 (Fig. 5F, Figure S6). Although OVV-mNbTIM3 cured 80% of the mice’s tumors, combination therapy with anti-PD-1 or anti-CTLA4 further promoted complete tumor remission in mice, achieving 100% or 90% remission rates respectively. (Fig. 5F, Figure S6). No weight loss was observed in mice treated with OVV, OVV-hNbTIM3 or combination therapy (Fig. 5G). This result indicates that TIM3 nanobody further enhances the ability of oncolytic viruses to promote the efficacy of immune checkpoint antibody-based cancer therapy.

### Efficacy of intratumoral immune responses in humanized mice and PDX model

**Fig. 6:**
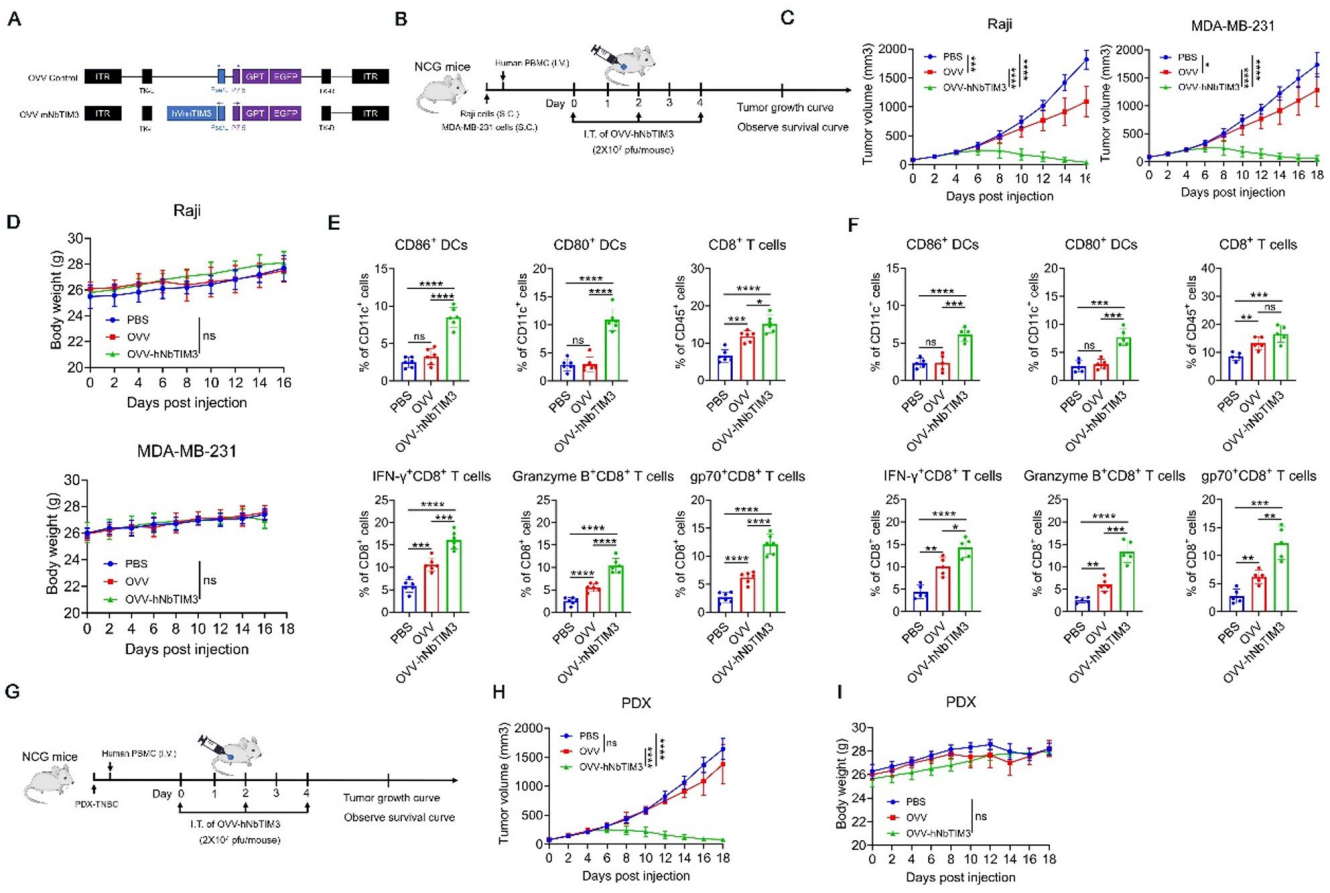
Antitumor activity of OVV-hNbTIM3 in vivo. **A** Schematic diagram of a recombinant oncolytic vaccinia virus expressing hNbTIM3. **B** Schematic representation of experimental design and treatment timeline. Raji or MDA-MB-231 tumor-bearing NCG mice were intravenously injected with human peripheral blood mononuclear cells, and then mice were administered OVV-hNbTIM3, OVV or PBS (*n* = 6). **C** Tumor growth. **D** Body weight. **E**-**F** Flow cytometric analysis of the proportions of CD80^+^ DCs, CD86^+^ DCs, CD8^+^ T cells, IFN-γ^+^CD8^+^ T cells, Granzyme B^+^CD8^+^ T cells and gp70^+^CD8^+^ T cells in Raji (**E**) or MDA-MB-231 (**F**) tumors. **G** Schematic representation of experimental design and treatment timeline. The PDX tumor cells were injected into the fourth mammary fat pads of NCG mice, and these tumor-bearing mice were intravenously injected with human peripheral blood mononuclear cells on day 1, and then mice were administered OVV-hNbTIM3, OVV or PBS (*n* = 6). **H** Tumor growth. **I** Body weight. Data are shown as the mean ± SD. ns, no significant difference; *p < 0.05, **p < 0.01, ***p < 0.001, ****p < 0.0001

Studies in immunocompetent mouse models demonstrated the marked effectiveness of OVV-mNbTIM3 against mouse tumors as described above in nonhumanized mice. To assess the effect of oncolytic activity and immune activation potential against human tumors, we engineered a vaccinia virus carrying an anti-human TIM3 single domain antibody gene (OVV-hNbTIM3) (Fig. 6A). Western blot analysis confirmed efficient expression of hNbTIM3 in OVV-hNbTIM3-infected Raji cells (Figure S7A). To validate binding functionality, culture supernatants from OVV-hNbTIM3-infected cells were incubated with human T cells, followed by anti-FLAG antibody staining. Flow cytometry demonstrated specific binding of secreted hNbTIM3 to surface TIM-3 on both human dendritic cells and CD8^+^ T cells (Figure S7B). Cytolytic activity assays revealed comparable dose-dependent oncolysis between OVV-mNbTIM3 and parental OVV in human tumor models, with no statistically significant differences observed in either crystal violet staining or MTT viability assays (Figures S7C-D). Furthermore, both viruses exhibited identical replication kinetics in tumor cell lines (Figure S7E). These findings collectively demonstrate that insertion of the hNbTIM3 transgene does not compromise the replication efficiency or oncolytic capacity of the recombinant virus.

To examine the ability of OVV-hNbTIM3 to change the tumor immune microenvironment and boost antitumor efficacy, we evaluated OVV-hNbTIM3 in tumor-bearing humanized mice into which human peripheral blood mononuclear cells (PBMC) had been grafted to model a human immune system (Fig. 6B). OVV control showed antitumor activity in this model, but OVV-hNbTIM3 demonstrated even higher efficacy and induced tumor regression (Fig. 6C). No weight loss was observed in mice treated with OVV-hNbTIM3 (Fig. 6D). Increased numbers of tumor-infiltrating mature DCs and activated CD8^+^ T cells were observed in mice treated with OVV-hNbTIM3 compared to mice treated with OVV control, either in Raji models (Fig. 6E) or MDA-MD-231 models (Fig. 6F). More importantly, OVV-hNbTIM3 treatment also inhibited tumor growth in the patient-derived xenograft (PDX) TNBC model (Figs. 6G-I). The activity of OVV-hNbTIM3 in the humanized mouse model is consistent with that of OVV-mNbTIM3 observed in immunocompetent mouse models, suggesting an important role for intratumoral expression of TIM3 antibody.

## Discussion

Our study reveals that OVV-induced immunosuppressive feedback mechanisms attenuate anti-tumor immunity within the TME. Specifically, OVV-infected tumor cells drive CD8^+^ T cells dysfunction characterized by an exhaustion phenotype. To address these limitations, we investigated whether TIM-3 blockade could mitigate CD8^+^ T cells exhaustion during OVV therapy, demonstrating that this combinatorial approach not only proves clinically feasible but also synergistically enhances anti-tumor immunity through TME remodeling. Building on these findings, we engineered a recombinant oncolytic vaccinia virus (OVV-NbTIM3) expressing single-domain antibodies targeting murine/human TIM-3, which effectively reprograms immunosuppressive TME landscapes toward pro-inflammatory phenotypes conducive to tumor clearance.

While OVV demonstrate unique potential as immunotherapeutic agents through TME reprogramming and immune activation [31], the compensatory immunosuppressive pathways induced during viral therapy remain poorly characterized. Here we show that although OVV treatment transiently converts “cold” tumors to “hot” immunogenic states, this response is counterbalanced by marked CD8^+^ T cells exhaustion in 4T1 and A20 murine models, thereby limiting therapeutic efficacy. Mechanistically, prolonged antigen exposure and chronic immune activation drive T cells dysfunction, characterized by upregulation of multiple inhibitory receptors (PD-1, TIM-3) coupled with profound epigenetic and transcriptional reprogramming. These alterations render exhausted CD8^+^ T cells incapable of effective tumor cell eradication despite initial activation.

Our previous studies demonstrated that combinatorial OVV and anti-PD-1 therapy synergistically enhances tumor immunotherapy efficacy [30, 31]. Notably, T cells co-expressing PD-1 and TIM-3 exhibit more profound dysfunction than those expressing PD-1 alone, with dual PD-1/TIM-3 blockade demonstrating superior capacity to restore exhausted T cells functionality compared to monotherapy [14]. While multiple anti-TIM-3 antibodies are currently under evaluation in clinical trials, therapeutic interpretation remains challenging given TIM-3’s expression across diverse immune subsets beyond CD8^+^ T cells [32, 33]. Our current investigation reveals that OVV-NbTIM3 exerts its primary effect through DCs reprogramming to drive potent anti-tumor immunity. This focus on DCs is particularly relevant given cDC1s’ established role in orchestrating effective anti-tumor immune responses and immunotherapy outcomes, as T cells checkpoint inhibition alone proves insufficient without cDC1-mediated antigen presentation to expand tumor-specific CD8^+^ T cells populations.

Although prior studies have explored oncolytic virus/anti-TIM3 combinations, including engineered herpes simplex virus YST-OVH with CTLA-4/TIM-3 blockade [34] and dual PD-1/TIM-3 inhibition in refractory lung cancer models [35], our study uniquely demonstrates that TIM3 nanobody-armed OVV significantly augments anti-tumor immune responses through dual mechanistic pathways. While direct TIM3 neutralization on CD8^+^ T cells partially restores effector functions, our findings unexpectedly highlight TIM-3 blockade on DCs as the dominant mechanism, dramatically enhancing antigen presentation capacity. This bidirectional interplay creates a self-reinforcing circuit wherein mature DCs sustain prolonged T cells activation, effectively overcoming adaptive immune resistance mechanisms within the tumor microenvironment [36]. We assumed that many pathogen-associated molecular patterns (PAMPs), damage-associated molecular patterns (DAMPs), and tumor associated antigens (TAAs) would emerge with OVV-mNbTIM3-mediated killing of tumor cells and these contents may continue to potentiate DCs phenotypic maturation. Several limitations warrant consideration in interpreting our findings. First, the absence of patient-derived specimens from ongoing OVV clinical trials precludes direct demonstration of the proposed immune feedback loop mediated by OVV therapy-a critical factor for clinical translation. Second, while our data underscore the pivotal role of TIM-3 expression on DCs in orchestrating robust anti-tumor immunity, the precise molecular mechanisms underlying OVV-NbTIM3-mediated DCs activation require further mechanistic dissection in future investigations.

In summary, OVV therapy induces functional impairment in DCs and exhaustion of CD8^+^ T cells, while anti-TIM-3 intervention effectively reprograms DCs toward immunostimulatory phenotypes, thereby augmenting T cells-mediated tumor control. These insights hold significant implications for rational design of combinatorial oncolytic viral therapies and bioengineered OV platforms. Notably, the translational relevance is supported by: [1] the established clinical use of vaccinia virus as a leading oncolytic agent; [2] the mature development pipeline of anti-TIM-3 antibodies in multiple cancer indications; [3] demonstrated therapeutic efficacy of this combination strategy across preclinical murine and patient-derived xenograft models; and [4] functional conservation of DCs/CD8^+^ T cells regulatory pathways between murine systems and human hematopoietic lineages.

## Supplementary Information

Supplementary Material 1.

## Data Availability

All data reported in this paper will be shared upon request.
