# Supplementary material for: TIM-3 blockade reverses oncolytic vaccinia virus-induced DCs inactivation and T cells exhaustion to improve antitumor immunity and therapeutic efficacy
Source: J Exp Clin Cancer Res. 2025 Nov 25;44:333. doi: 10.1186/s13046-025-03596-0 (PMC12751143; doi:10.1186/s13046-025-03596-0)
Supplement: Supplementary file 1 — Supplementary Material 1. [file 13046_2025_3596_MOESM1_ESM.docx]

**Supplementary Figures S1-S7 and legends**


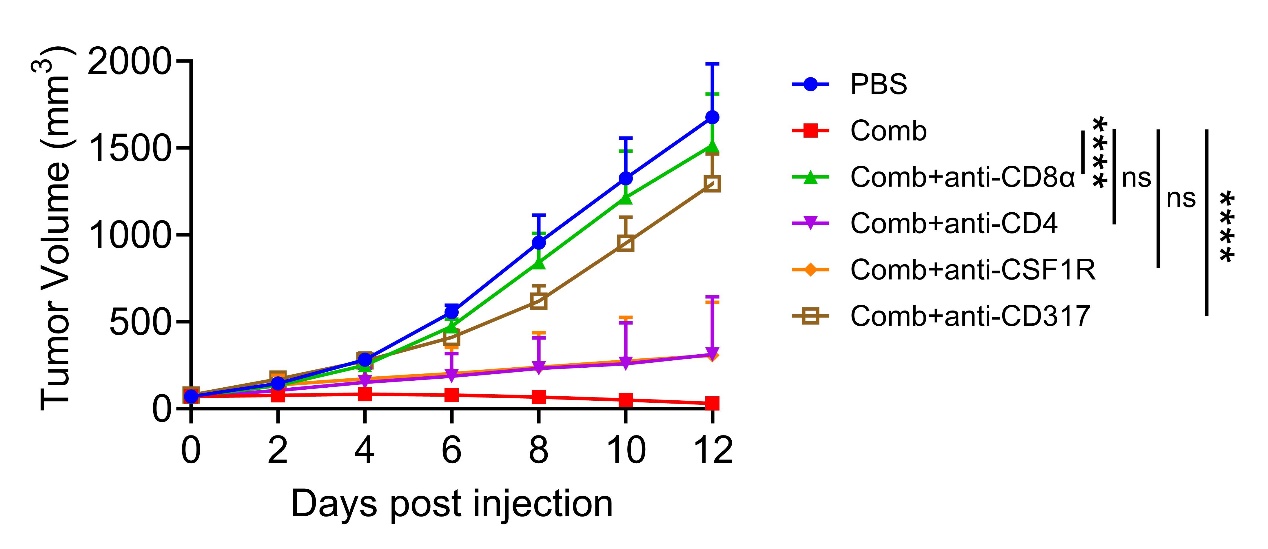


Figure S1. DCs and CD8^+^ T cells mediate the antitumor activity of combination therapy

A20 tumor-bearing mice were administered combination therapy and treated with intraperitoneal injections of anti-CD8α, anti-CD4, anti-CSF1R or anti-CD317. Tumor volume was measured every 2 days during a 12 day-period (n = 6 biological replicates). The data are shown as the means ± SD. ns, no significant difference; ****p < 0.0001.


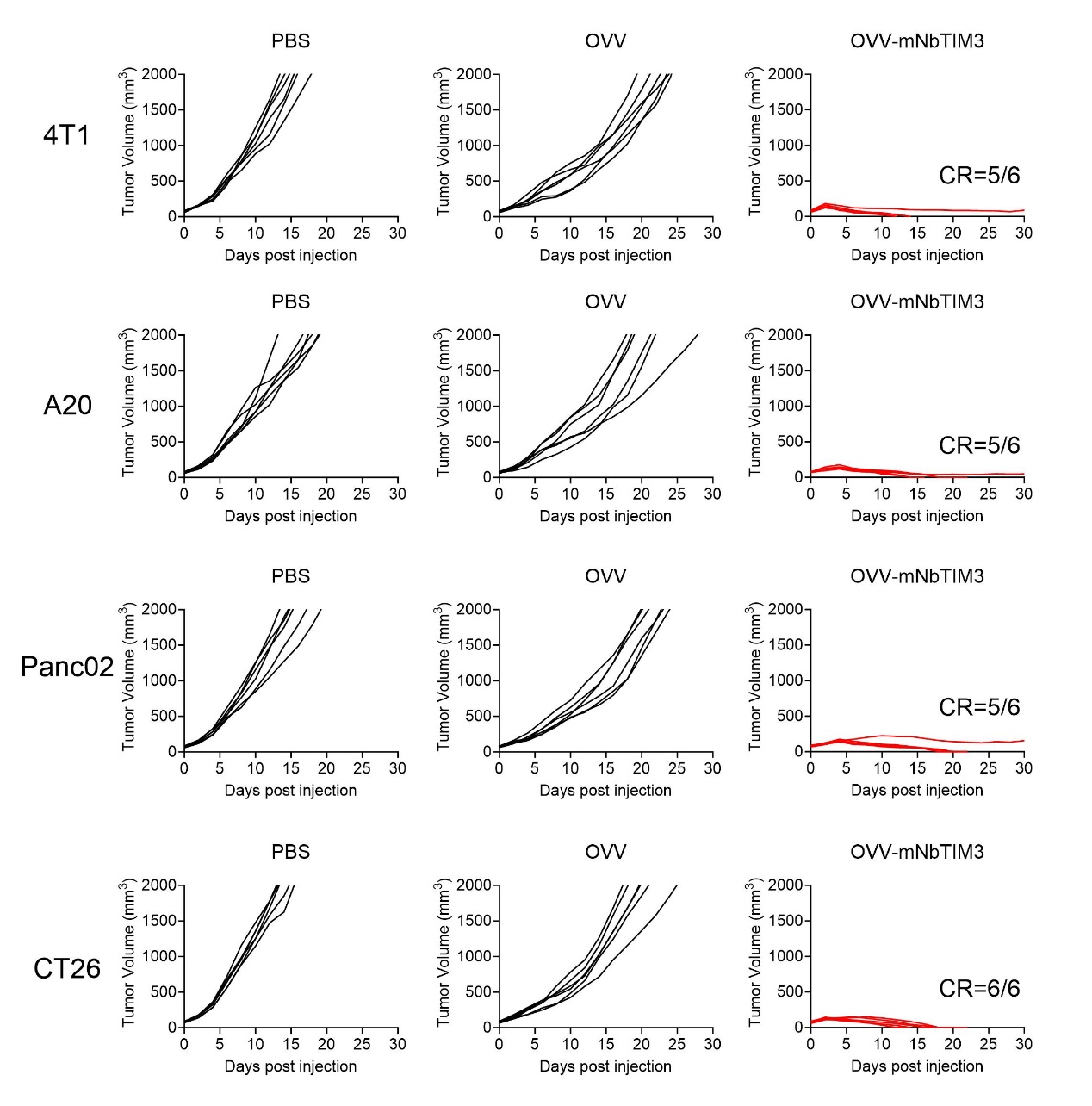


Figure S2. Individual tumor growth curve of mice. The tumor volume was measured every two days. Once the tumor volume of the mouse exceeded 2000 mm^3^, it was no longer measured. CR: complete remission.


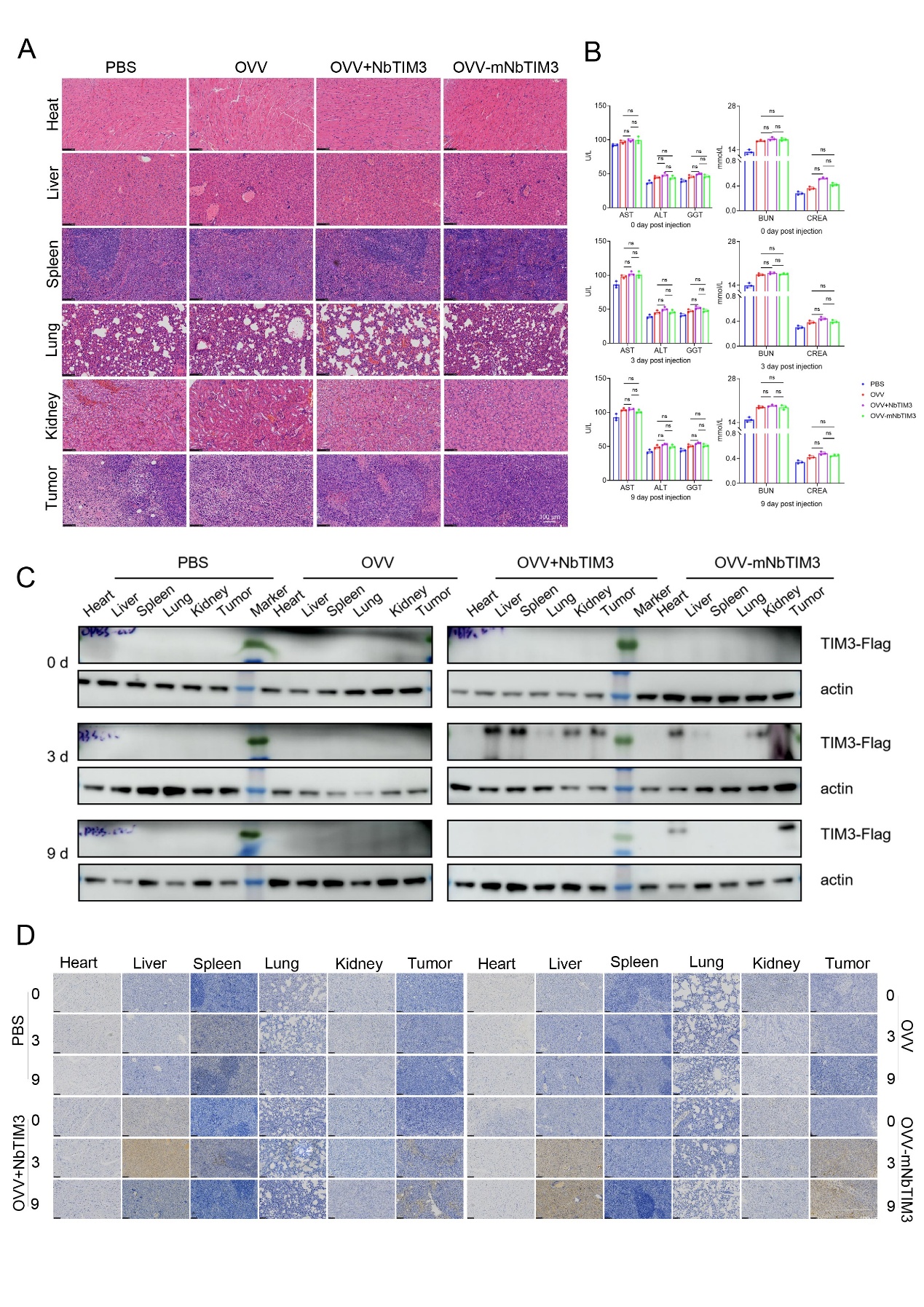
Figure S3. Distribution and biosafety assessment of mNbTIM3. (A) HE staining of major organs in mice. (B) Serum blood routine test. (C) Western blot analysis of the distribution of mNbTIM3 in mice. (D) IHC analysis of the distribution of mNbTIM3 in mice. Error bars represent SD. ns, not significant; **** p < 0.0001.


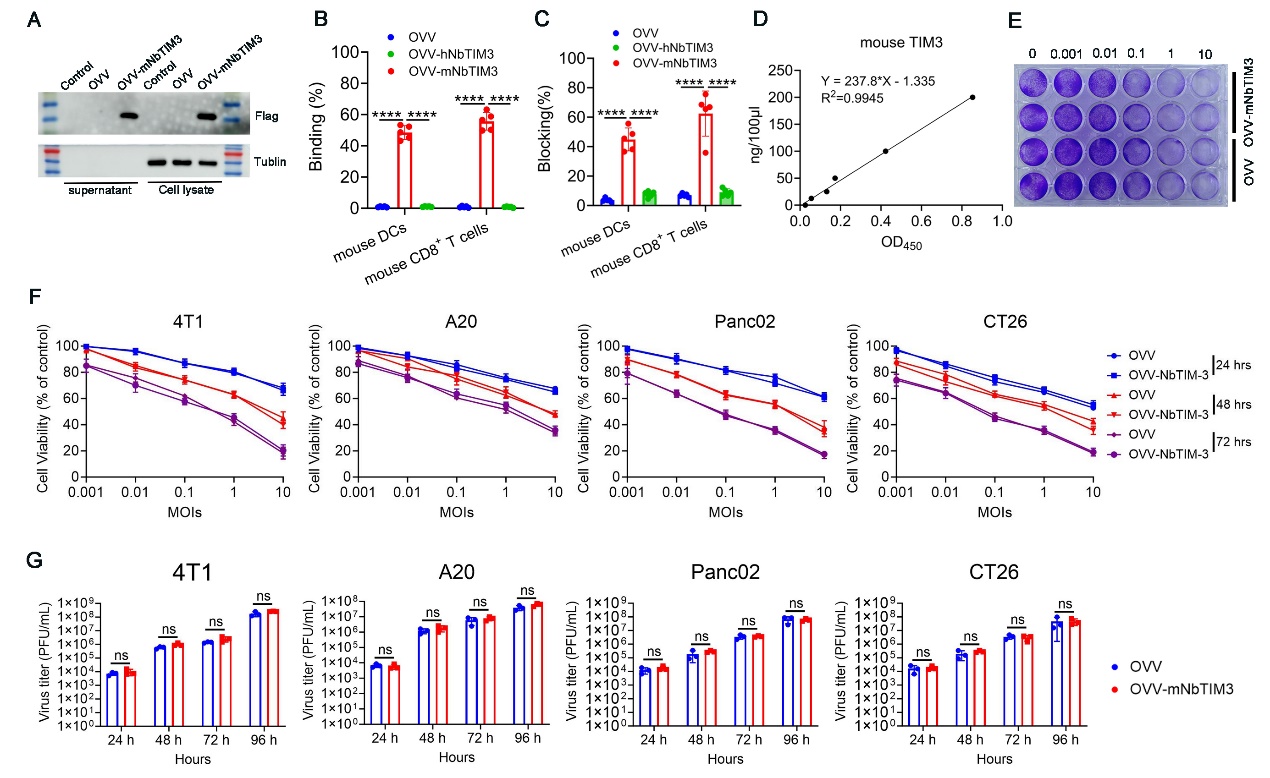


Figure S4. Characterization of OVV-mNbTIM3. (A) Western blot analysis of OVV-mNbTIM3-induced mNbTIM3 overexpression in A20 murine tumor cells. (B-C) DCs and CD8^+^ T cells were sorted out and incubated with OVV/OVV-mNbTIM3/OVV-hNbTIM3 supernatants. The binding (B) and blocking (C) of mNbTIM3 was detected by flow cytometry. (D) Mouse TIM3 proteins were coated on the ELISA plates, mNbTIM3 protein (purified from cell-free system) was tittered as standard sample and the curve was fitted. (E) Crystal violet staining was used to detect the oncolytic ability of OVV-mNbTIM3 and OVV control against 4T1 murine tumor cells. (F) MTT assay was used to detect the oncolytic ability of OVV-mNbTIM3 and OVV control against murine tumor cells. (G) TCID50 method was used to detect viral replication in murine tumor cells. Error bars represent SD. ns, not significant; **** p < 0.0001.


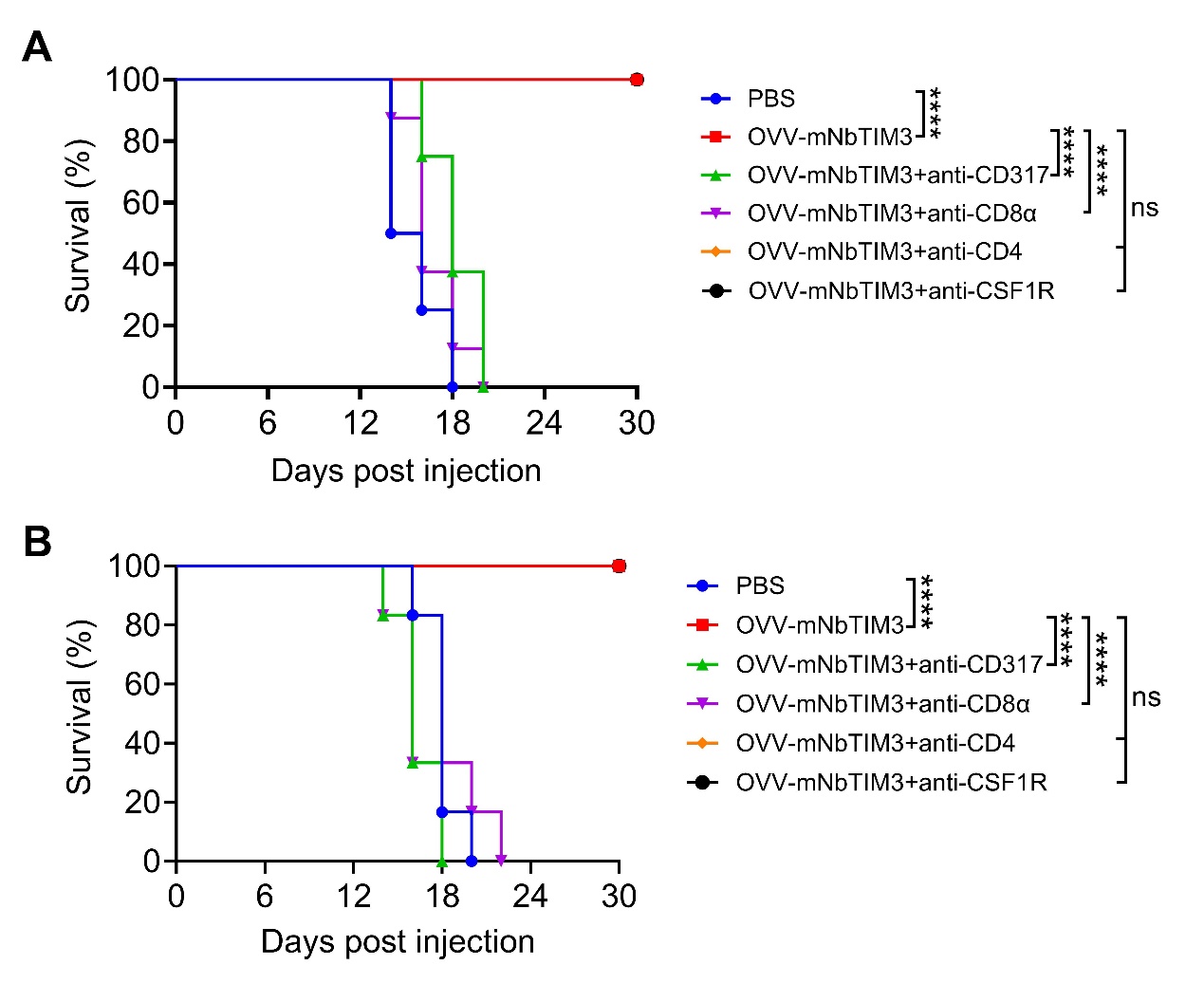


Figure S5. DCs and CD8^+^ T cells mediate the antitumor activity of OVV-mNbTIM3 therapy (A) A20 or (B) 4T1 tumor-bearing mice were administered OVV-mNbTIM3 and treated with intraperitoneal injections of anti-CD8α, anti-CD4, anti-CSF1R or anti-CD317. Tumor volume was measured every 2 days during a 30 day-period (n = 8 biological replicates for A20; n = 6 biological replicates for 4T1). The data are shown as the means ± SD. ns, no significant difference; ****p < 0.0001.


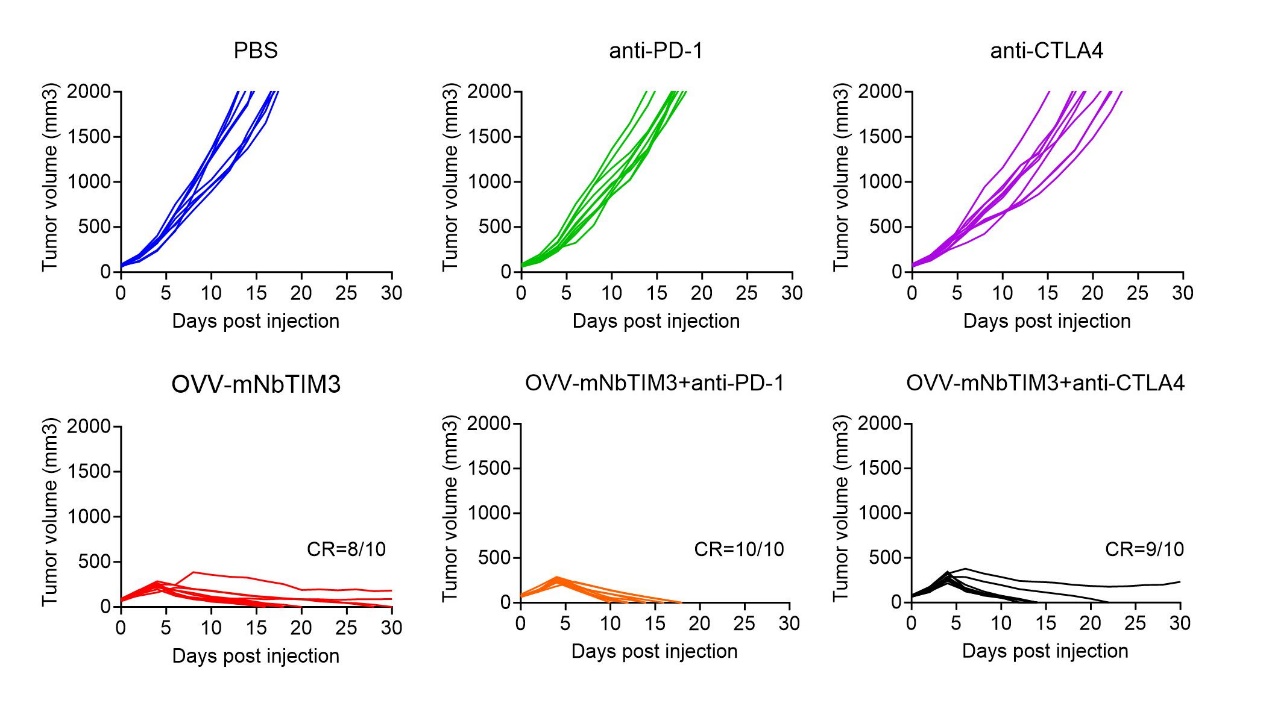


Figure S6. Individual tumor growth curve of combination therapy of OVV-mNbTIM3 and anti-PD-1 antibody or anti-CTLA4 antibbody. The tumor volume was measured every two days. Once the tumor volume of the mouse exceeded 2000 mm^3^, it was no longer measured. CR: complete remission.


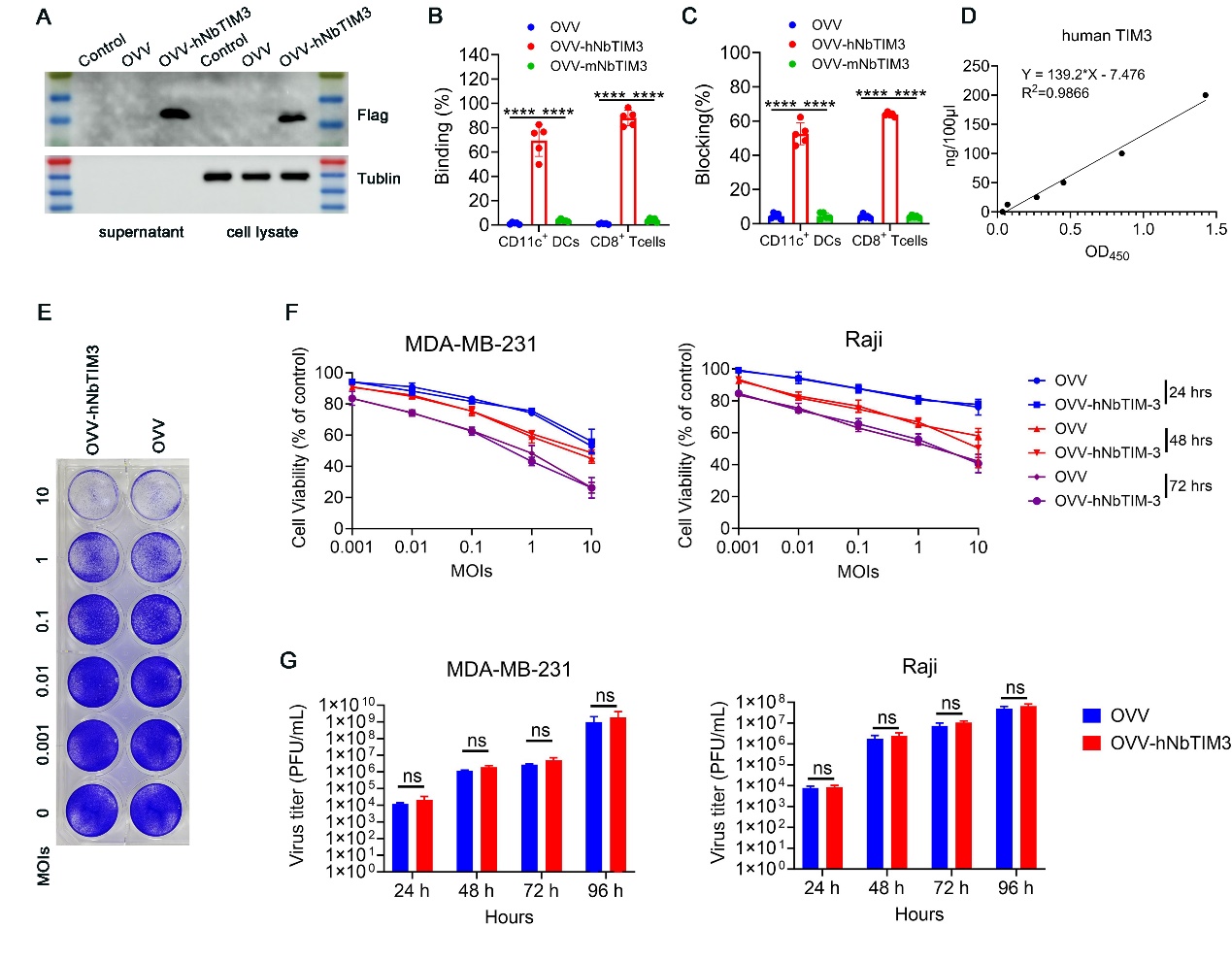


Figure S7. Characterization of OVV-hNbTIM3. (A) Western blot analysis of OVV-hNbTIM3-induced hNbTIM3 overexpression in Raji human tumor cell line. (B-C) DCs and CD8^+^ T cells were sorted out and incubated with OVV/OVV-hNbTIM3/OVV-mNbTIM3 supernatants. The binding (B) and blocking (C) of hNbTIM3 was detected by flow cytometry. (D) Human TIM3 proteins were coated on the ELISA plates, hNbTIM3 protein (purified from cell-free system) was tittered as standard sample and the curve was fitted. (E) Crystal violet staining was used to detect the oncolytic ability of OVV-hNbTIM3 and OVV control against MDA-MB-231 murine tumor cells. (F) MTT assay was used to detect the oncolytic ability of OVV-hNbTIM3 and OVV control against human tumor cells. (G) TCID50 method was used to detect viral replication in human tumor cells. Error bars represent SD. ns, not significant; **** p < 0.0001.
